# Protecting effect of emodin in experimental autoimmune encephalomyelitis mice by inhibiting microglia activation and inflammation via Myd88/PI3K/Akt/NF-κB signalling pathway

**DOI:** 10.1080/21655979.2022.2052671

**Published:** 2022-04-07

**Authors:** Kenan Zheng, Baojiang Lv, Lulu Wu, Chen Wang, Haoyou Xu, Xiaojun Li, Zhibing Wu, Yuanqi Zhao, Zequan Zheng

**Affiliations:** a The First Clinical School, Guangzhou University of Chinese Medicine, Guangzhou, China; b Lingnan Medical Research Center, Guangzhou University of Chinese Medicine, Guangzhou, China; c Department of Traditional Chinese Medicine, Zhujiang Hospital, Southern Medical University, Guangzhou, China; d Department of Neurology, The Second Affiliated Hospital of Guangzhou University of Chinese Medicine, Guangdong Provincial Hospital of Traditional Chinese Medicine, Guangzhou, China; e The Second Clinical School, Guangzhou University of Chinese Medicine, Guangzhou, China; f Department of Neurology, The First Affiliated Hospital of Guangzhou University of Chinese Medicine, Guangzhou, China; g Doctor of equivalent degree, Guangzhou University of Chinese Medicine, Guangzhou, China

**Keywords:** emodin, MS/EAE, PI3K/AKT/NF- κB signalling pathway, TLRs/MyD88 signal, microglia activation, myelin protective, network pharmacology

## Abstract

Experimental autoimmune encephalomyelitis (EAE) is characterized by demyelination of the central nervous system. Emodin is an anthraquinone derivative with comprehensive anti-inflammatory, anti-cancer, and immunomodulatory effects and is widely used in the treatment of inflammatory, tumor, and immune system diseases. However, none of the clinical or experimental studies have explored the therapeutic efficacy of emodin in EAE/multiple sclerosis (MS). Thus, we evaluated the protective effect of emodin on EAE mediated via inhibition of microglia activation and inflammation. Wild-type mice were randomly divided into the normal control, EAE, low-dose emodin, and high-dose emodin groups. Clinical scores and pathological changes were assessed 21 days after immunization. The network pharmacology approach was used to elucidate the underlying mechanisms by using an online database. Molecular docking, polymerase-chain reaction tests, western blotting, and immunofluorescence were performed to verify the network pharmacology results. An *in vivo* experiment showed that high-dose emodin ameliorated clinical symptoms, inflammatory cell infiltration, and myelination. Pharmacological network analysis showed AKT1 was the main target and that emodin played a key role in MS treatment mainly via the PI3K–Akt pathway. Molecular docking showed that emodin bound well with PI3K, AKT1, and NFKB1. Emodin decreased the expression of phosphorylated(p)-PI3K, p-Akt, NF-κB, and myeloid differentiation factor 88 and the levels of markers (CD86 and CD206) in M1- and M2-phenotype microglia in EAE. Thus, the emodin inhibited microglial activation and exhibited anti-inflammatory and neuroprotective effects against EAE via the Myd88/PI3K/Akt/NF-κB signalling pathway. In conclusion, emodin has a promising role in EAE/MS treatment, warranting further detailed studies.

## Introduction

Multiple sclerosis (MS) is an inflammatory demyelinating disease of the central nervous system (CNS) with unclear pathogenesis. Environmental variables and genetic factors fundamentally contribute to MS. Because of the lack of effective treatments, disability caused by relapse and progression of the disease not only brings physical and mental distress to patients but also virtually adds tremendous burden to families and society [1,2].

Experimental autoimmune encephalomyelitis (EAE) is characterised by demyelination of the CNS and is useful in understanding the pathological mechanism of MS. Autoimmune responses induced by CD4^+^ T cells are considered a pathogenic factor throughout the EAE course. This process includes an imbalance of T-cell subsets, inflammatory cascade, cell necrosis, and apoptosis [3]. Although adaptive immunity plays a crucial role in the process, innate immunity is also indispensable in the acute stage of the disease. Evidence shows that microglia are involved in inducing an immune-inflammatory response in the CNS; in the acute stage of EAE/MS, microglia migrate to the lesions and immediately acquire the pro-inflammatory phenotype (M1). By expressing MHC molecules, microglia stimulates brain-derived T-cell production, pro-inflammatory cytokine production, and subsequently, inflammation and CNS demyelination. EAE/MS involves the infiltration of myelin-reactive CD4^+^ T helper (Th) cells and the release of pro-inflammatory cytokines, which lead to inflammatory infiltrates, demyelination, and axonal damage in the CNS [4]. During disease progression, the migration of autoreactive T cells across the blood–brain barrier increases [5]. Inflammation and neurodegeneration are independent process occurring in MS as well as in EAE brain [6]. Toll-like receptor (TLR) is a response molecule of innate immunity that plays a key role in microglia activation and mediates inflammatory responses, with TLR4 signalling being well-known among these responses. TLR4 signalling activates the microglia by triggering a myeloid differentiation factor 88 (MyD88) or Toll/IL-1 R domain-containing adaptor, inducing the IFN-β (TRIF, also known as ticam1)-dependent signalling pathway, and activating the downstream PI3K/Akt signalling pathway [7,8]. After phosphorylation, PI3K/Akt can further activate NF-κB, thereby causing the initiation of inflammatory cascade reactions, which might cause damage to the tissues [9,10]. Therefore, regulating microglia differentiation and inhibiting inflammatory responses in the CNS are essential in EAE treatment.

The treatment principle of relapsing MS involves short-course steroid therapy of 3–5 days to relieve symptoms and to initiate disease-modifying therapy as early as possible [11,12]. However, steroids and immunomodulators have therapeutic limitations and several side effects [13,14]. Therefore, discovering novel therapeutic drugs is important. Natural products such as emodin having remarkable effectiveness have garnered considerable scientific attention. Modern pharmacology has revealed that emodin exhibits good anti-inflammatory and immunomodulatory activities in various diseases such as tumors, immune diseases, and neurodegenerative diseases [15,16]. Thus, we believe that emodin exerts potential therapeutic effects on EAE/MS.

Network pharmacology is a newly emerging field based on system biology and is considered a promising approach in pharmaceutical research. It can be used to study the interaction among several compounds, targets, and pathways of active components to elucidate the underlying mechanisms of their action [17]. By studying the interaction between drug action components and disease targets, a complex drug–target interaction network can be constructed using this approach [18]. Therefore, network pharmacology is widely used for identifying disease targets and biological functions and predicting molecular mechanisms underlying various diseases.

We aimed to find protective effects of emodin on EAE/MS by inhibiting the microglia activation and inflammation via TLR signalling pathway. The present study explored the role of emodin in the treatment of EAE/MS by using network pharmacology and further verified the mechanism of action of emodin by which myelin oligodendrocyte glycoprotein (MOG) induced EAE in mice. This study can provide an experimental reference for natural product treatment of EAE/MS.

## Materials and methods

We evaluated the amelioration of clinical and pathological changes of emodin against EAE/MS. Network pharmacology was used to screen the core targets of emodin against EAE/MS, the interaction network between compounds and screening targets established via the intersection of drug targets and disease targets and enrichment to find out their biological pathways and explain their therapeutic mechanisms. The completion of the experiment will provide a scientific basis for the action of emodin on EAE and its clinical application. Molecular docking, polymerase chain reaction tests, western blotting, and immunofluorescence were performed to explore the mechanism of emodin against EAE/MS.

## Reagents and chemicals

MOG 35–55 (MEVGWYRSPFSRVVHLYRNGK, purity >98%) was synthesized by China Peptides Co., Ltd, Shanghai, China). Complete Freund’s adjuvant (CFA) was purchased from Sigma-Aldrich (St. Louis, MO, USA), Mycobacterium tuberculosis strain H37Ra (MTB) was purchased from BD Biosciences (San Diego, USA), and Pertussis toxin (PTX) was purchased from List Biological Labs (California, USA). Emodin (NO:A0044, purity >98%) was purchased from Chengdu Must Bio-Technology Co., Ltd, China (Chengdu, China), sodium carboxymethyl cellulose was purchased from Shanghai Aladdin Biochemical Technology Co., Ltd. (Shanghai, China). Emodin was dissolved in sodium carboxymethyl cellulose (sample: solvent, 1:200, m/v). Antibodies specific for CD86 (DF6332), GAPDH (AF7021) and Ticam1 (DF6289) were purchased from Affinity Biosciences Co., Ltd. (Changzhou, China). Anti-CD206 (ab64693), anti-myelin-basic protein (MBP) (ab218011), anti-brain-derived neurotrophic factor (BDNF) (ab108319), anti-phosphorylated(p)-Pi3k(ab191606), anti-p-AKT (ab81283), anti-MyD88 (ab219413), anti-NF-κB p50 (ab32360), Alexa Fluor 488 AffiniPure Donkey Anti-Goat IgG(H + L) (ab150129) and Goat Anti-Rabbit IgG H&L (HRP) (ab6721) were purchased from Abcam (Cambridge, USA). Anti-Iba1(011–27,991) was provided by FUJIFILM Wako Pure Chemical Corp. (Osaka, Japan). Peroxidase AffiniPure Goat Anti-Rabbit IgG (H + L) (111–035-003) was provided by Jackson ImmunoResearch Laboratories, Inc (Pennsylvania, USA). Hematoxylin eosin (HE) staining kit (SBJ-0446) was provided by SenBeiJia Biological Technology Co., Ltd, China (Nanjing, China), Alexa Fluor 555 AffiniPure Donkey Anti-Rabbit IgG (H + L), antibodies specific for PI3K (AF7742), AKT1 (AF1777), TLR4 (AF7017), and HRP-labeled Goat Anti-Rabbit IgG (H + L) (A0208) were provided by Beyotime Biotechnology Co., Ltd (Shanghai, China). RNA Easy Fast Tissue/Cell RNA Extraction Kit (DP451), FastKing gDNA Dispelling RT SuperMix (KR118), and Talent qPCR PreMix (SYBR Green) (FP209) were provided by TIANGEN BIOTECH (BEIJING) CO., LTD. (Beijing, China)

## Animals housing and management and emodin preparation

All the animal care and experimental procedures were approved by Institutional Animal Ethics Committee of the First Affiliated Hospital of Guangzhou University of Chinese Medicine (Ethics No. TCMF1-2,020,008). Healthy specific pathogen-free C57BL/6 wild-type (WT) female mice were purchased from Guangdong Medical Laboratory Animal Center (Licence No. SCXK (Guangdong) 2018–0002). The mice were aged 6–8 weeks and weighed 18–20 g. All were kept in standard cages in a controlled specific pathogen-free environment with free access to standard rodent food and tap water at 23 ± 3°C with 40–60% humidity and 12-h light/dark cycle light control.

## EAE model induction and animal behavior test

Firstly, the mice were randomly divided into the normal control (NC) group, EAE group, low-dose emodin group (Emodin-L), and high-dose emodin group (Emodin-H) (n = 9 in each group). The mice of EAE and emodin groups received subcutaneous injections of 200 µg MOG 35–55 emulsified 1:1 in CFA containing 4 mg/mL heat-killed MTB on day 0, followed by 500 ng PTX intraperitoneally on days 0 and 2 to induce the EAE [19,20]. Next, NC and EAE mice were treated with sodium carboxymethyl cellulose. While the mice of Emodin-L and Emodin-H groups were treated with 30 mg/kg/d and 60 mg/kg/d emodin, respectively. The treatment by oral gavage started at day 0 days post immunization (dpi). Then, test animal behavior and pathological changes by HE and IHC of BDNF and MBP. Second, choose the best dose of emodin group to have further investigation.

The neurological clinical behavioral scores were applied as a method of animal behavior test in the experiment daily, in a masked manner, using the following established standard [21]: 0, no signs of disease; 1, loss of tail tonicity; 2, loss of tail tonicity and mild paralysis of hindlimbs; 3, paralysis of hindlimbs; 4, hindlimb paralysis and mild paralysis of forelimbs; and 5, complete paralysis or death. On 21 dpi, all mice were sacrificed by using 2% pentobarbital sodium intraperitoneal injection to anesthetize deeply. At the same time, their brains and lumbar spinal cords were collected and kept for further studies.

## Hematoxylin-eosin (HE) staining and immunohistochemistry (IHC)

After 21 dpi, slices of brain and lumbar spinal cords were fixed in 4% paraformaldehyde for 24 h. Then, following Dehydration, embedding, and dewaxing, the slices were stained by H-E for pathological and inflammation observation. The inflammatory parameters were based on a 5-point scale as described previously [22]: 0, no sign of inflammation; 1, scattered inflammatory cells; 2, some inflammatory cells and karyopyknosis; 3, perivascular inflammatory cell infiltrate; and 4, marked inflammatory cell infiltration into the parenchyma.

As for IHC, slices of brain were repaired by citrate rinsed, washed in PBS, permeabilized in hydrogen peroxide at room temperature for 10 min, and incubated with primary antibodies at 4°C for 12 h. Following washed in PBS, secondary antibody Peroxidase AffiniPure Goat Anti-Rabbit was applied at 37°C for 30 min. Then, slices were washed in PBS and stained by DAB. Hematoxylin re-stained by 3–5 min, fraction sections by alcohol and hydrochloric acid, gradient alcohol dehydration, xylene transparency, air-drying, and neutral gum sealing. The following primary antibodies were used: myelin basic-protein (MBP) and brain-derived neurotrophic factor (BDNF).

Observe the slices of lumber spinal cords under the inverted fluorescence microscope IX-73 (Olympus Corporation, Tokyo, Japan) and slices of brain under PANNORAMIC MIDI II Digital Slide Scanners (3D HISTECH, Budapest, Hungary). Five non-overlapping fields of view were randomly selected in each slice and the inflammation scores were evaluated. Slices of brain were seen in Caseviewer system (Budapest, Hungary). The IHC results were expressed as IOD values by Image pro plus 6.0.

## Drug-likeness prediction

To evaluate pharmacokinetics, drug-likeness, and medicinal chemistry friendliness of small molecules, the SMILES format CC1 = CC2 = C(C(=C1)O)C(=O)C3 = C(C2 = O)C = C(C = C3O)O of emodin was input into the SwissADME tool (http://www.swissadme.ch). Next, screening was performed under the default parameters.

## Emodin-related targets prediction

To find putative targets of emodin, Encyclopedia of Traditional Chinese Medicine (ETCM, http://www.tcmip.cn/ETCM/index.php/Home/Index/index.html) [23], SwissTargetPrediction (http://www.swisstargetprediction.ch) [24], Pharmmapper (http://www.lilab-ecust.cn/pharmmapper/) [25] and HERB (http://herb.ac.cn/) [26]databases were used. Then, UniProt (https://www.uniprot.org) [27] was used to normalize the naming of the drug targets. All targets were selected along with other default arguments.

## MS-related targets prediction

To identify the known targets of MS, Database resources of GeneCard (https://www.genecards.org, Relavance ≥ 10) [28], DisGeNET (https://www.disgenet.org/, score ≥ 0.05) [29], and OMIM (https://omim.org/) [30] were obtained. These MS-related targets were mapped with STRING (https://www.string-db.org) [31] to the human standard gene ID. Meanwhile, ‘Relapsing Remitting Multiple sclerosis’, ‘Secondary Progressive Multiple Sclerosis’, ‘Multiple Sclerosis, Primary Progressive’ and ‘Progressive Relapsing Multiple Sclerosis’ were used as keywords in DrugBank (www.drugbank.ca/) database [32]. The common targets (both MS- and Emodin-related) were shown in the Venn Diagram (http://bioinformatics.psb.ugent.be/webtools/Venn/) and kept for further network construction and analysis.

## PPI network and molecular complex module analysis tool(MCODE) analysis of emodin in the treatment of MS

The PPI network map was constructed for the curated databases, experimental determined, co-expression, fusion, neighborhood, and co-localization of potential target genes with predicted gene interactions [33]. The common targets were put into the STRING database where the organism was set as ‘Homo sapiens’, and The PPI network with a medium confidence score > 0.4 was selected and imported into Cytoscape software version 3.8.2 for interaction network visualization [34–37]. The topological parameters of each node, including the degree centrality (DC), betweenness centrality (BC), closeness of centrality (CC) and local average connectivity-based method (LAC) were evaluated by CytoNCA plug-in in Cytoscape software. And the degree is more than 2.0 point. The PPI network for emodin and MS was constructed and then clustered through MCODE plugin in Cytoscape software. The core targets were further screened and by their degree centrality (DC) values. The information of each node was calculated to derive Score values and functional modules.

## Enrichment analysis

All the common targets were imported into the Metascape (https://metascape.org/gp/index.html) [38] for enrichment analysis. The Gene Ontology (GO) and KEGG enrichment analysis results were corrected by applying FDR methods. FDR < 0.01 was accepted to obtain main GO and KEGG pathways, and the first 30 pathways were visualized. Afterward, Omicshare online tools (https://www.omicshare.com/tools/) were used to screen out and analyze the enrichment of GO and KEGG pathways for potential targets.

## Molecular docking studies

To verify whether the emodin has binding force with the targets, the X-ray crystal structures of symbol genes selected from KEGG enrichment were obtained from the Protein Data Bank (PDB, https://www.rcsb.org/) [39]. Then, AutoDockTools (version 1.5.6, http://autodock.scripps.edu/) was used to perform molecular docking and calculate the binding affinity to confirm the lower the binding energy, standing for the stable-binding force. The docking results of active compounds and protein targets were visualized with PyMOL software (version 2.2, https://pymol.org/2/)

## Immunofluorescence

Slices of brains were repaired by citrate rinsed for 30 min and washed in PBS 3 times for 5 min each after dehydrating. After dewaxing to water, firstly, the paraffin sections were put into xylene, anhydrous ethanol, and alcohol (concentration of 95%, 90%, 80%, 70%, each for 5 min) then washed by running water and distilled water. Secondly, EDTA repair for 3 min, slowly cool to room temperature for 30 min. Third, rinsed in PBS 3 times for 5 min each. Fourth, circle the tissue with an immunohistochemistry pen. Fifthly, close the sections by sheep serum at room temperature for 1 h. Then, primary antibody anti-CD86 and anti-incubated at 4 degrees for 12 h. After PBS (1–3 cylinders) washed for 3–5 min, use secondary antibody to incubate sections at 37 degrees for 60 min. To visualize the nuclei, DAPI blocker to seal the slice. The following primary antibodies were used: CD86 and CD206.

Observe the slices under the inverted fluorescence microscope (IX-73, Olympus Corporation, Tokyo, Japan) and PANNORAMIC MIDI II Digital Slide Scanners (3D HISTECH, Budapest, Hungary). Five non-overlapping fields of view were randomly selected in each slice and the inflammation scores were evaluated. Immunohistochemically stained images were seen with the Caseviewer system. Results were expressed as integrated optical density (IOD) values by Image pro plus 6.0.

## Real-time polymerase chain reaction(RT-PCR) assay

Total RNA was isolated from cortex tissues using RNA Easy Fast Tissue/Cell RNA Extraction Kit. First-strand cDNA synthetized by FastKing gDNA Dispelling RT SuperMix following the RT-PCR performed by using SYBR Green in the ABI7500 Real-Time PCR Detection System (Thermo Fisher Science, Waltham, Massachusetts, USA). All steps were performed according to the manufacturer’s instructions. All primers sequences were listed in (Table 1). Results were analyzed using 2^−ΔΔCt^ method.

**Table 1.** PCR primers

Genes	Forward sequence (5’ to 3’)	Reverse sequence (5’ to 3’)
IL-6	AGCCAGAGTCCTTCAGAGAGA	GCCACTCCTTCTGTGACTCC
TGF-β	AGGGCTACCATGCCAACTTC	CCACGTAGTAGACGATGGGC
RORγt	CAGAGACACCACCGGACATC	CCCAGATGACTTGTCCCCAC
IL-17A	CAACCGTTCCACGTCACCCT	CCAGCTTTCCCTCCGCATT
TLR4	TGGCTGGTTTACACGTCCAT	TGCAGAAACATTCGCCAAGC
Myd88	AAGCAGCAGAACCAGGAGTC	CGAAAAGTTCCGGCGTTTGT
Ticam1	GGGATCGGTGCAGTTCAGAT	TGGTGTGTCAATGGGACGAG
GAPDH	CATCACTGCCACCCAGAAGACTG	ATGCCAGTGAGCTTCCCGTTCAG

## Western blot

The homogenates isolated from the cortex tissues were centrifuged at 13,000 rpm for 15 minutes at 4°C. Protein concentration was determined by the BCA method. Protein extracts were separated by SDS-PAGE gels and transferred onto PVDF transfer membranes. The films were sealed with 5% nonfat dry milk powder dissolved in Tris-buffered saline and Tween-20 (TBST) at room temperature for 1 h. Then, the membranes were incubated with the primary antibodies pi3k (1:5000), p-pi3k (1:1000), Akt (1:1500), p-Akt (1:5000), TLR4 (1:1000), Myd88 (1:1000), Ticam1 (1:1000), and NF-κB p50 (1:5000) overnight. After being washed with TBST for 3 times, the membranes were incubated in HRP-labeled goat anti-rabbit (1:10,000) secondary antibody for 1 hour at room temperature. The membranes were washed with TBST for 3 times, and the color was detected with ECL chemiluminescence solution and scanned with chemiluminescence system.

## Statistical analysis

Data analyses were performed using SPSS 25.0 software (IBM, Armonk, NY, USA). Two-group comparisons were made using the Mann-Whitney U test or student’s t-test, while three groups were compared by one-way ANOVA or Kruskal-Wallis test using Bonferroni comparisons post hoc tests. Data are shown as mean ± standard deviation (S.D). *P* < 0.05 were deemed statistically significant.

## Results

### Emodin ameliorated the clinical scores and reduced the pathological changes in the brains and spinal cords of EAE

**Figure 1.**
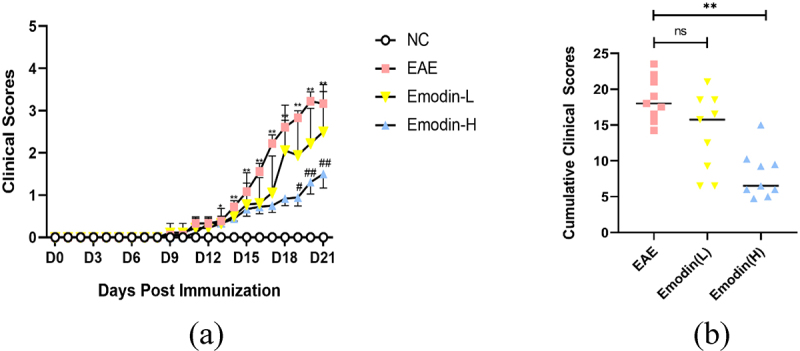
Emodin ameliorated the EAE clinical scores. (a) Clinical scores changes in NC, EAE, and emodin on 21 dpi. (b) Cumulative clinical scores of two groups on 21 dpi. Data were expressed as means ± standard deviation (n = 9). Comparisons among four groups were analyzed by one-way ANOVA on 21 dpi in A. Comparisons among four groups were analyzed by Kruskal-Wallis test using Bonferroni comparisons post hoc tests except 21 dpi in A. Comparisons among EAE, emodin-L, and emodin-H groups were analyzed by the one-way ANOVA in B. *P < 0.05, **P < 0.01 versus NC group, ^#^*P* < 0.05, ^##^*P* < 0.01 EAE group, and ‘ns’ stands for not significance.

The symptoms such as loss of tail tonicity, staggering gait, hind-limb paralysis, four-limb paralysis, and even death appeared sequentially in EAE mice [40]. To investigate the therapeutic effect of emodin in EAE, EAE was induced with MOG35-55 in C57BL/6 mice. The daily clinical scores of animals and cumulative scores were calculated. The first clinical score of EAE mice appeared around 11 dpi and increased in the following days. Compared to NC mice, the clinical score on 11–21 dpi significantly increased in EAE mice (*P* < 0.05, Figures 1a). Compared with EAE mice, emodin-L group demonstrated a tendency for decreasing clinical scores daily and there was statistically significance between emodin-H and EAE groups in 21 dpi (Figures 1a). Additionally, the cumulative scores of the groups of emodin-L and emodin-H also were decreased compared to the EAE mice. Significant decrease was shown in the groups of Emodin-H (Figures 1b).

**Figure 2.**
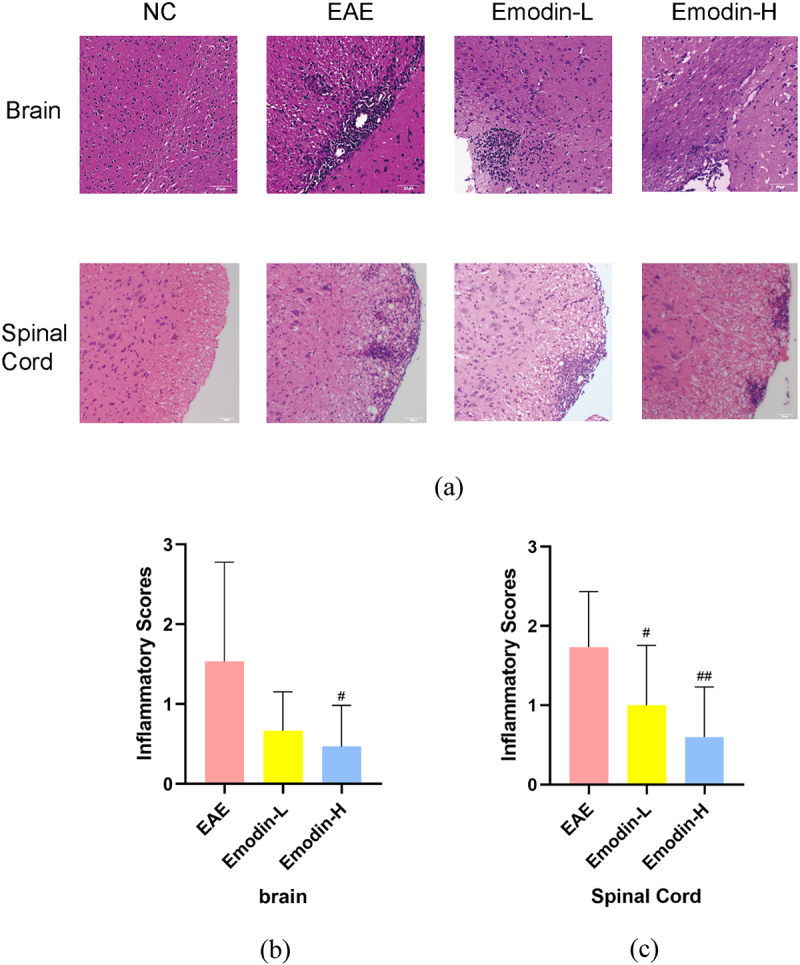
The pathological changes showed that emodin ameliorated inflammatory infiltration under the HE staining in the brains and spinal cords of mice on 21 dpi of EAE (a). The pictures were taken at ×200 magnification. The inflammatory scores of brains (b) and spinal cords (c) among EAE, emodin-L, and emodin-H groups on 21 dpi. Data expressed as means ± S.D (n = 3). Comparisons among three groups were analyzed by Kruskal-Wallis test using Bonferroni comparisons post hoc tests. ^#^*P* < 0.05 versus EAE group, ^##^*P* < 0.01 versus EAE group.

All mice were sacrificed on 21 dpi, and the brain and the spinal cord were collected for pathological analysis. Infiltration of inflammatory cells can evaluate the pathological changes of EAE by HE staining. The marked inflammatory cell infiltration was observed in EAE mice. Inflammatory cells as perivascular cuffs infiltrating around small blood vessels were seen in the brain and spinal cords of EAE mice. The inflammatory cell Infiltration of spinal cords and brain in the emodin-H groups is lighter than the EAE group in statistical significance (*P* < 0.05, Figures 2). Emodin-L showed no significant decrease compared to EAE group (Figures 2). These results indicated that high-dose emodin had the potential to ameliorate EAE.

## Emodin alleviates demyelination and promotes remyelination

**Figure 3.**
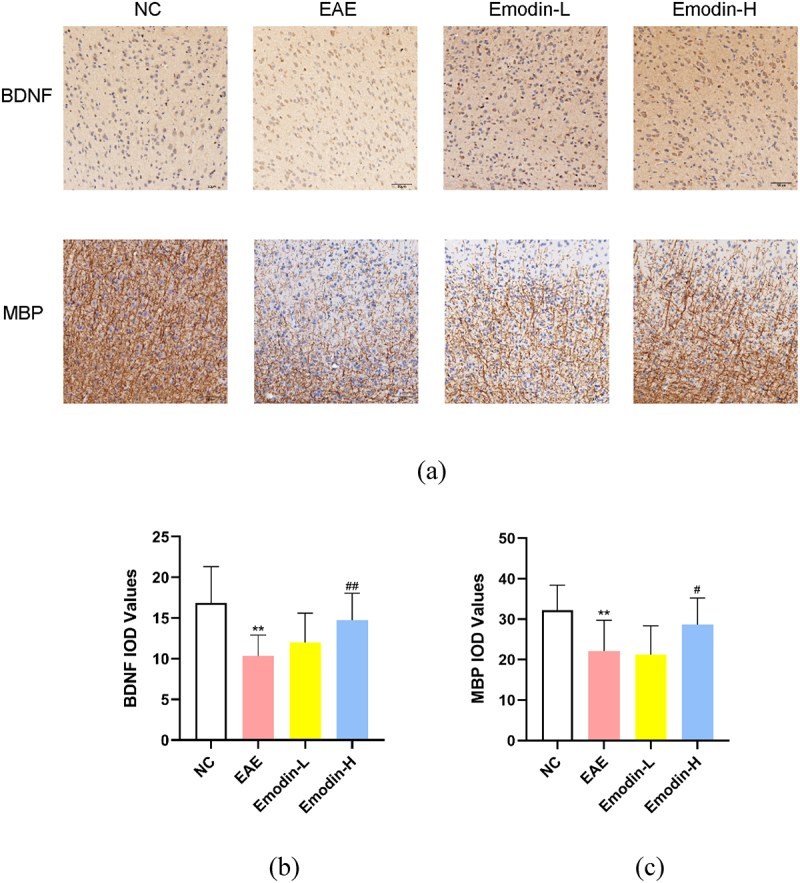
The IHC staining of MBP and BDNF in the brains of mice on 21 dpi of EAE (a). The pictures were taken at ×200 magnification. The inflammatory scores of brains (b) and spinal cords (c) among NC, EAE, emodin-L. and emodin-H groups on 21 dpi. Data expressed as means ± S.D(n = 3). Comparisons among four groups were analyzed by one-way ANOVA in B and C. ^#^*P* < 0.05 versus EAE group, ^##^*P* < 0.01 versus EAE group.

MS pathology features the focal demyelinating lesions in the CNS. MBP and BDNF can be detected to evaluate the remyelination [41,42]. Based on the clinical and inflammatory infiltration amelioration of emodin on EAE, the remyelination effect of emodin in EAE was examined. The IHC was employed to detect the IOD of MBP and BDNF(Figures 3a). Lower BDNF and MBP IOD expression was found in the EAE model group than NC group (*P* < 0.01, Figures 3b,c). Emodin-H enhanced the MBP and BDNF IOD expression levels in the brain of EAE mice significantly (*P* < 0.05, Figures 3b,c) while no significant change was found in the Emodin-L group (Figures 3b,c). The results showed that high-dose of emodin improved the myelination of EAE mice.

## Molecule properties of emodin

**Figure 4.**
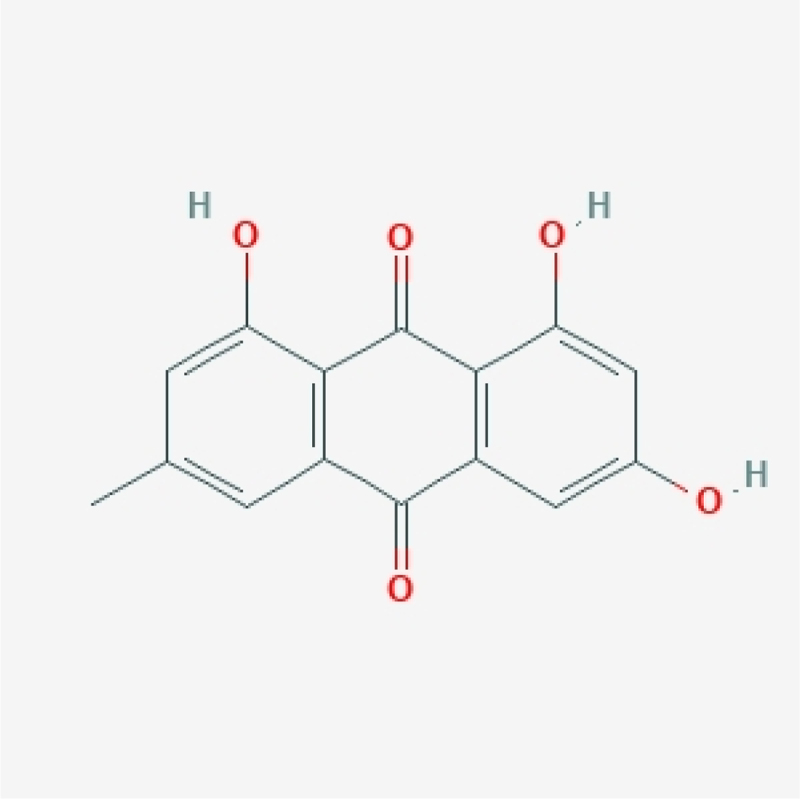
The 2D structure of emodin.

In order to explore the mechanism of emodin in the treatment of EAE, we evaluated the basic information of the molecular structure of the natural product. According to Lipinski’s RO5, the MV of a drug-like compound should be less than 500 g/mol, the topological polar surface area (TPSA) less than or equal to 140 A^2^, the calculated XLogP3 less than 5, the rotatable bond less than 10, the hydrogen bond acceptors no more than 10, and the hydrogen bond donors no more than 5. Through the SwissADME database and ETCM, The properties of emodin were screened in line with the RO5 and drug-likeness weight, indicating that it had good drug-like properties (Table 2). The structure of emodin was shown (Figures 4).

**Table 2.** Molecule properties of emodin

Property	Value
Drug-likeness weight	0.683
Molecule weight	270.24 g/mol
TPSA	94.83 A^2^
XlogP3	2.72
H-bond donor	3
H-bond acceptor	5
Molar refractivity	70.78
Lipinski	Yes
Bioavailability score	0.55

## Target identification results and analysis

**Figure 5.**
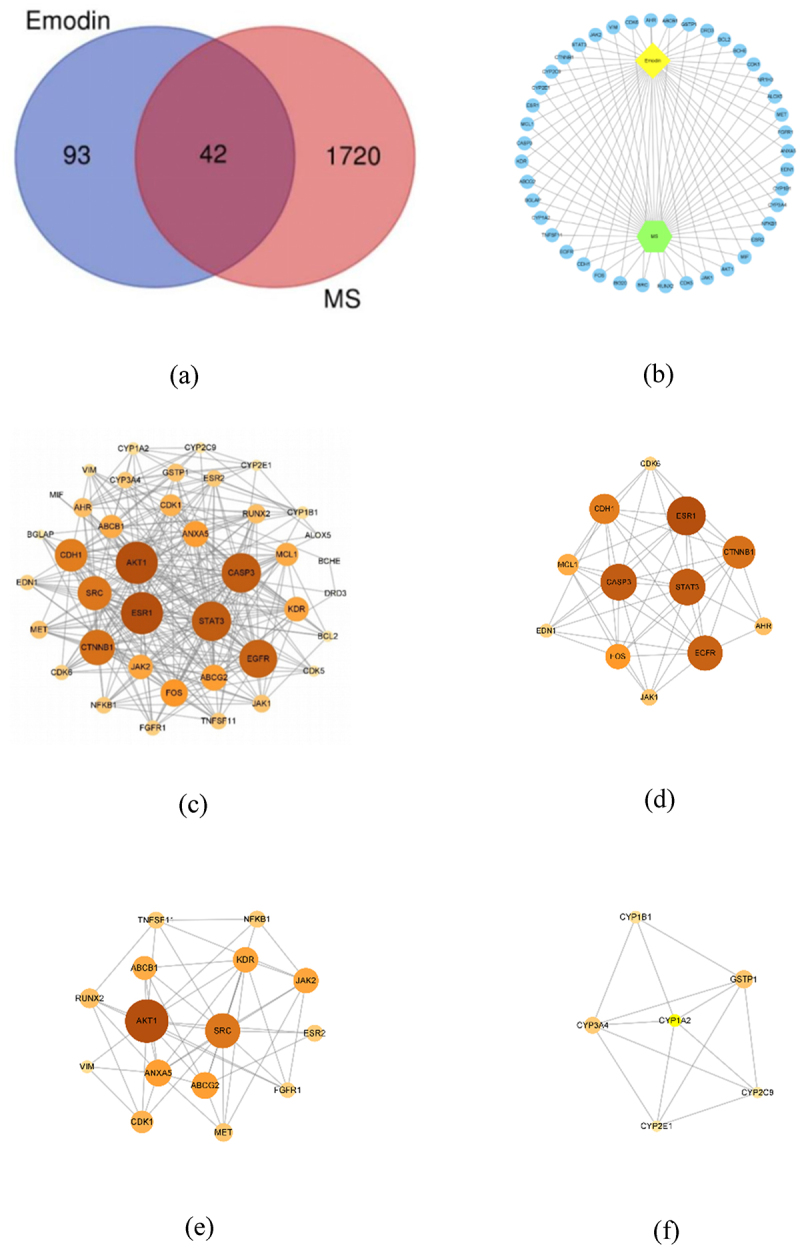
The network diagram and the PPI network construction. Venn diagram of common targets showing emodin-related and MS-related common targets (a). The network diagram of emodin-targets-disease network was constructed (b). The PPI network was visualized by cytoscape 3.8.2 software (c). The larger node area and the darker color marked the most important targets in the network. The MCODE interaction network for visualization in cytoscape 3.8.2 software which divided into module 1 (d), module 2 (e), and module 3 (f).

Through all database, 137 of emodin and 1762 MS-related genes were found. Among them, there were 42 common related genes considered as potential core genes (Figures 5a). The Emodin-Targets-Disease network diagram was visualized which show that emodin could have effects on multiple sclerosis by multiple targets (Figures 5b).

## PPI network construction and MCODE analysis

In order to find the core regulatory genes and their functions, first, the collected 42 potential hub genes were put into STRING to construct PPI network. There were 40 nodes and 296 edges in the PPI network. Next, we imported it into CytoNCA plug-in in Cytoscape software. Ranking by DC, the top 10 targets were AKT1, ESR1, CASP3, STAT3, EGFR, CTNNB1, SRC, CDH1, FOS, and ANXA5 (Figures 5c). Through the MCODE plugin in Cytoscape software, the PPI network was clustered more than 2 points. Three MCODE networks identified were screened in Figures 5 and the scores were 8.909, 6.857, and 5.2, respectively. Ranking by DC and LAC, in module 1 (Figures 5d), ESR1, CASP3, STAT3, EGFR, CTNNB1, FOS, and CDH1 were main targets. In module 2 (Figures 5e), SRC, AKT1, ANXA5, KDR, and ABCG2 were main targets. In module 3 (Figures 5f), CYP1A2, CYP3A4, GSTP1 were main targets. Among them, AKT1, CASP3, STAT3, EGFR, FOS, CTNNB1, and CDH1 are related to cell differentiation, migration, proliferation, and apoptosis. AKT1, SRC, ANXA5, and KDR are mainly involved in inflammatory response. CYP1A2, CYP3A4, and GSTP1 are important biological enzymes for drug metabolism and detoxification. This suggests that emodin may participate in the treatment of EAE/MS by affecting cellular processes and regulating inflammatory response.

## KEGG and GO enrichment Analysis

**Figure 6.**
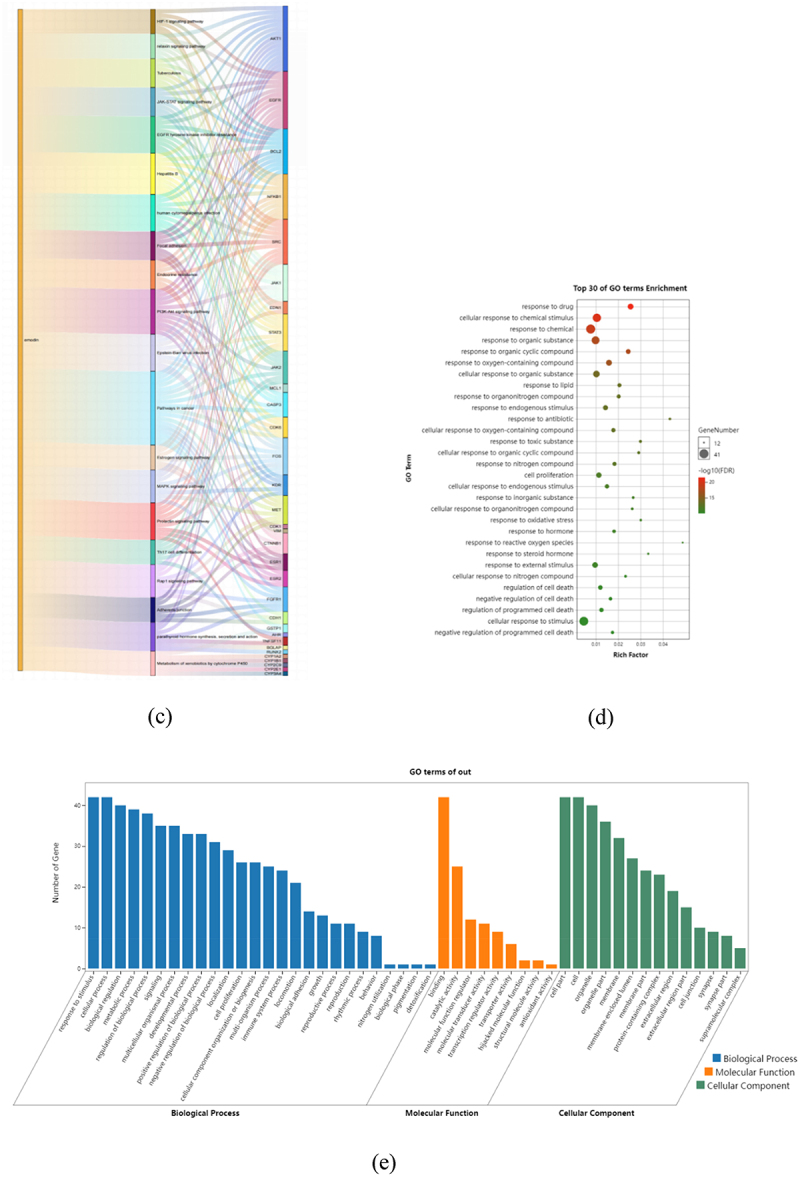
(Continued).

To investigate the mechanism of multitarget and multi-pathway of emodin’s effects on MS, the collected 42 core genes were utilized to analyze the pathways through KEGG and visualized through Omicshare online tools. In order to investigate the enrichment relationship between MS and these pathways, the KEGG pathway annotation was classified into six categories (Figures 6a). Ranking by the number of enriched genes, the top three systems that emodin might affect are endocrine, immune, and nervous systems, respectively. From the perspective of disease, cancer, infectious diseases, and drug resistance are the three main diseases. Immune and neurodegenerative diseases are also mentioned. In other classifications, biological processes involving more than 10 genes also include cell signal transduction, cellular community eukaryotes, cell growth, and death. These evidences suggest that emodin has great therapeutic potential for neurological and immune system diseases and may participate in the intervention process of diseases by regulating cell signalling transduction, affecting cell growth and apoptosis.
**Figure 6.**
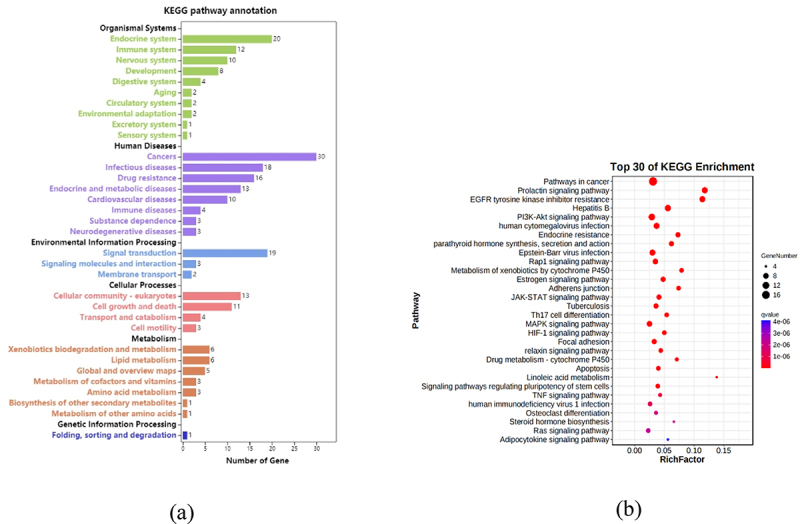
KEGG and GO enrichment analysis based on 42 core targets. Through KEGG pathway annotation (a), bubble chart (b), and Sankey diagram (c) of the pathways enriched for emodin and targets. GO enrichment analysis through bar chart (d) and bubble chart (e).

The KEGG enrichment indicated that these core genes were enriched in 85 pathways related to MS (FDR < 0.001 and term was set as ‘hsa’), summarized in 14 categories. The top five KEGG pathways with high counts including pathways in cancer, prolactin signalling pathway, EGFR tyrosine kinase inhibitor resistance, Hepatitis B pathway, PI3K/Akt signalling pathway (Figures 6b). Further analysis of these pathways showed that AKT1 is a key gene in the pathways mentioned above (Figures 6c). As an important member of the PI3K/Akt pathway, it participates in many links such as cell proliferation, survival, metabolism, and apoptosis. According to PPI and KEGG analysis, we believed that PI3K/Akt signalling pathway is more likely to participate in emodin intervention in EAE.

To investigate the biological roles of emodin for MS treatment, GO biological process (BP), cellular component (CC), and molecule function (MF) enrichment were analyzed and visualized by Omicshare online tools. As suggested in the results, the targets of emodin on MS were mainly related to the response to drug, cellular response to chemical stimulus, response to chemical stimulus, response to chemical, response to organic substance, response to organic cyclic compound, response to oxygen-containing compound, cellular response to organic substance, response to lipid and response to organonitrogen compound (Figures 6d). Furthermore, in the BPs, emodin had effects on the process of response to stimulus, cellular process, biological regulation, and biological process (Figures 6e), specifically including the response to drug, oxidative stress, and the response of reactive oxygen species for BPs. In the CCs, emodin targeted for cell part, organelle, membrane, specifically including the membrane raft, membrane microdomain, and membrane region. Meanwhile, the effects of emodin on MS were related to binding, catalytic activity, molecular function regulator, transcription regulator activity in the manners of the aromatase activity, protein kinase activity, protein tyrosine kinase activity, and heme binding at the level of GO MFs.

## Molecule docking

**Figure 7.**
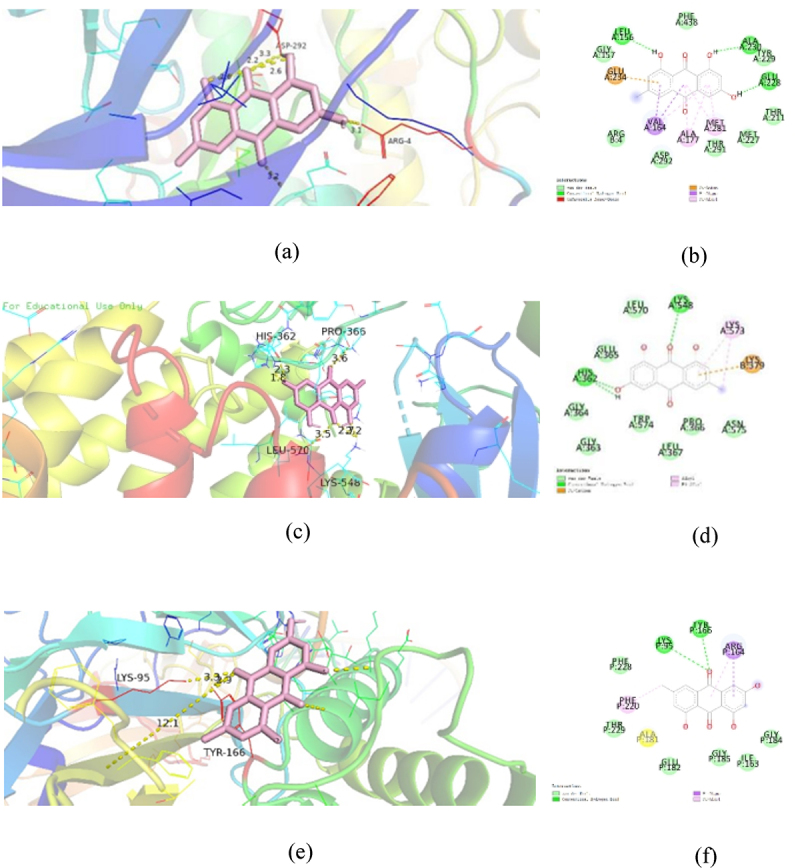
Docking models of emodin to symbol targets AKT1 (a), PI3K (b), and NFKB1 (c) in three-dimension. The emodin was shown in pink. All proteins are shown in ribbon model, and the color is red for amino acid codes. The hydrophobic interactions are displayed as yellow dashed lines. The two-dimension diagrams of AKT1 (d), PI3K (e), and NFKB1 (f) were shown.

To explore the binding force between emodin and symbol targets, molecular docking studies were carried out to confirm the interaction between emodin and related potential target genes at the molecular level. After molecular docking, emodin was found to bind well with AKT, PI3K, and NFKB1 (Table 3). The three-dimensional and two-dimensional maps of molecular docking images were shown (Figure 7). It is suggested that emodin had potential therapeutic effects on EAE through AKT, PI3K, and NFKB1.

**Table 3.** Docking scores of the active compounds of emodin with AKT1, PI3K, and NFKB1

Compounds	targets	Binding energy score (Gb:K cal/mol)	PDB ID	Combining with the residue (two-dimensional map)
Emodin	AKT1	−10.4	3MVH	ALA-230, GLU-228, LEU-156,GLU-234,MET-281,ALA-177,VAL-164
	PI3K	−9.0	4L23	HIS-362, PRO-366, LYS-548, LEU-570
	NFKB1	−7.1	1SVC	TYR-166, LYS-95,

## Emodin downregulated PI3K/Akt signal in EAE mice

Since previous study shows that high-dose emodin had significant effects of amelioration of clinical symptoms, inflammatory infiltration and remyelination on EAE. Low-dose emodin was not effective in reducing symptoms and inflammatory infiltration. Consequently, the group of emodin-H was applied to further investigation.

**Figure 8.**
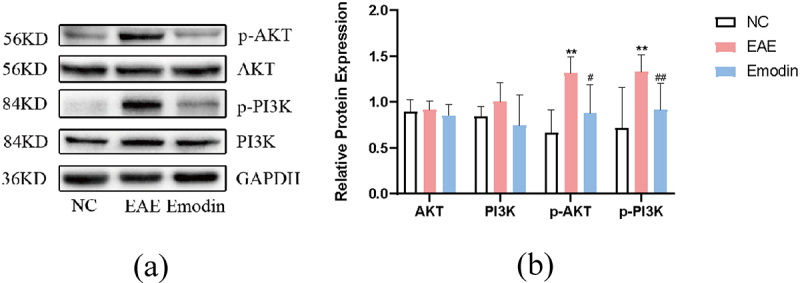
Effect of emodin on AKT, p-AKT, PI3K, and p-PI3K protein expression in cerebral cortex tissues on 21 dpi. The protein expression of AKT, p-AKT, PI3K, and p-PI3K was detected by western blot as described in the Materials and Methods. Data are represented as the mean ± S.D (n = 6). Comparisons among each group were analyzed by one-way ANOVA for detecting AKT, p-AKT, PI3K, and p-PI3K. *P < 0.05, **P < 0.01 versus NC; ^#^*P* < 0.01 ^##^*P* < 0.01, versus EAE.

In order to verify whether emodin treated EAE via PI3K/Akt signalling pathway which was enriched from the previous results of network pharmacology, the protein expressions of Akt, p-Akt, PI3K, and p-PI3K in cerebral cortex tissues were detected by western blot *in vivo*. The data showed that Akt and PI3K protein expressions were augmented in EAE. Emodin descended the Akt and PI3K protein expressions with no statistical significance (*P* > 0.05, Figures 8). Regardless of this result, p-Akt and p-PI3K protein expressions were significantly enhanced in the cortex tissue in EAE on 21 dpi compared to that in the NC mice (*P* < 0.01, Figures 8). while the treatment with emodin significantly diminished p-Akt and p-PI3K protein expressions (*P* < 0.05, Figures 8). We found that emodin could downregulate PI3K/Akt signalling by suppressing their phosphorylation, which suggested a potential mechanism in modulating cell proliferation of EAE. It is consistent with the result of the network pharmacology.

## Emodin ameliorated EAE by suppressing the microglia activation

**Figure 9.**
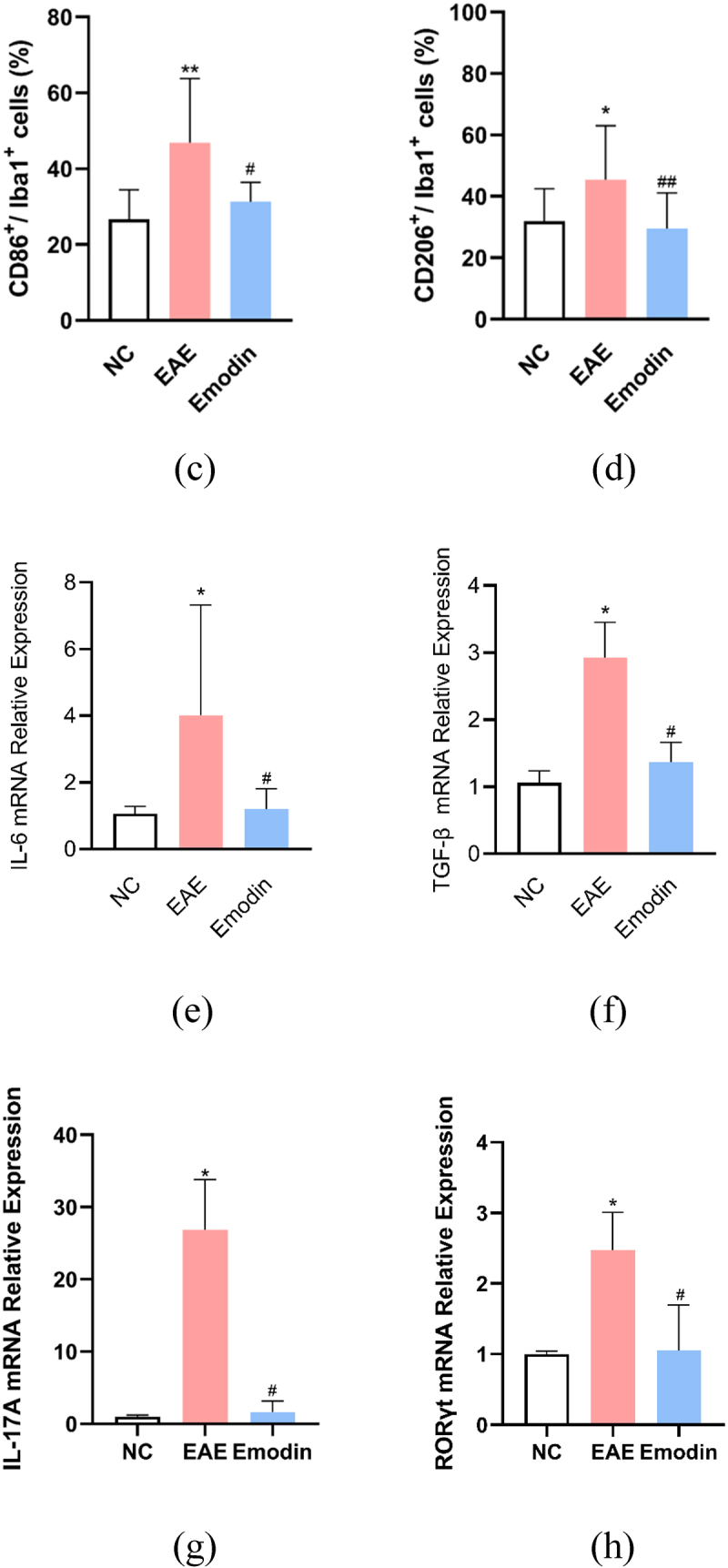
(Continued).

Then, we further investigated the effect of high-dose emodin on the activation of microglia in EAE to evaluate the innate immune state of CNS. The IF was employed to detect the number of CD86 and CD206, respectively, standing for the markers of M1 and M2 phenotype microglia (Figures 9a,b). The IF results in the CNS tissues of mice showed the expression levels of CD86 and CD206. Higher CD86 and CD206 expressions were found in the EAE model group than NC group and emodin inhibited both CD86 and CD206 expression of EAE mice significantly (*P* < 0.05, Figures 9c,d).
**Figure 9.**
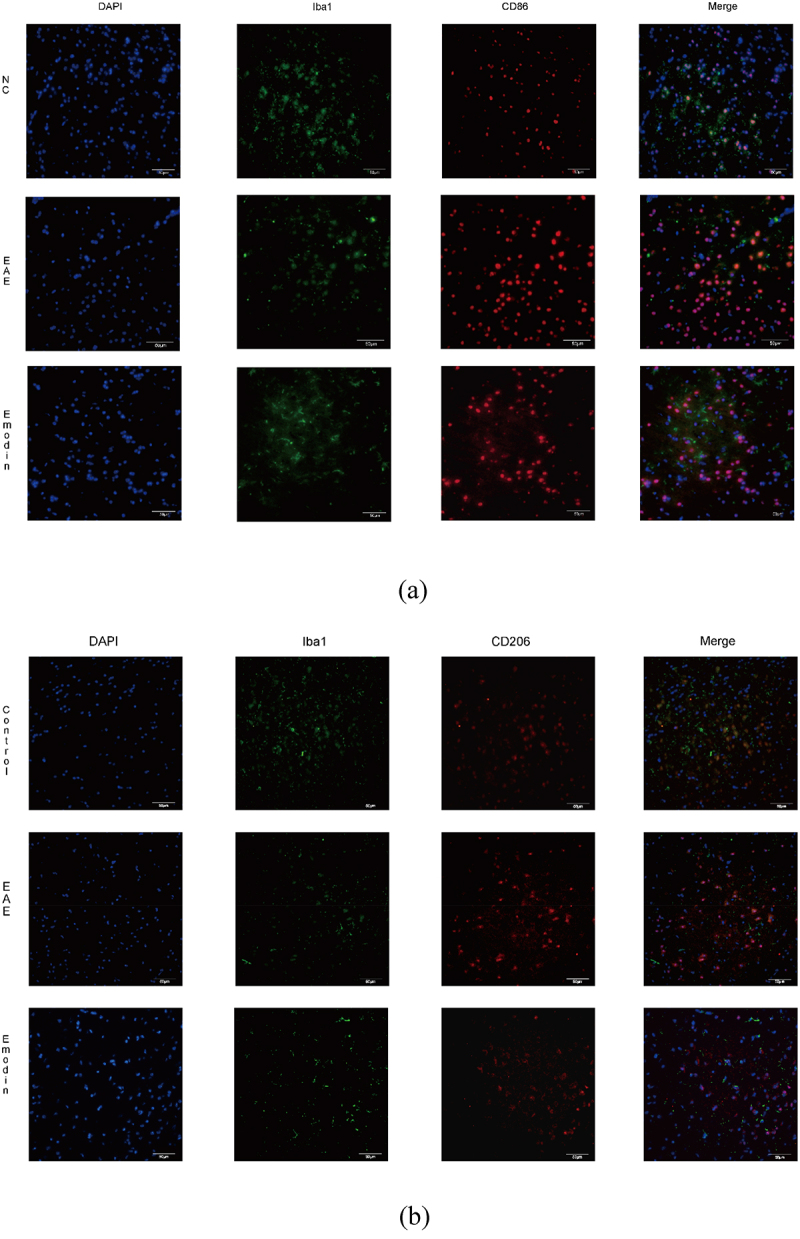
Emodin contributed to inhibition of the microglia activation. Double immunofluorescence staining of brain tissue with M1(CD86^+^/iba1^+^) (a) and M2(CD206^+^/iba1^+^) (b). The representative double immunofluorescence labeling image of CD86 (red)/Iba1 (green) in brain tissue, and the nuclei were stained with DAPI (blue). The representative double immunofluorescence labeling image of CD206 (red)/Iba1 (green) in brain tissue and the nuclei were stained with DAPI (blue). The quantity of the percentage of CD86^+^/Iba1^+^ cell (c) and CD206^+^/Iba1^+^ cell. (d) The effects of emodin on M1 microglia cell subsets mRNA relative expression. Mice were sacrificed on 21 dpi. The CNS tissues are collected. The IL-6 (e), TGF-β (f), IL-17A (g), and RORγt mRNA relative expression were analyzed by qRT-PCR, respectively. All data were expressed as mean ± S.D (n = 5). Comparisons among each group were analyzed by one-way ANOVA in C, D, F, and G. Comparisons among each group were analyzed by Kruskal-Wallis test using Bonferroni comparisons post hoc tests in E and H. *P < 0.05, **P < 0.01 versus NC group, ^#^*P* < 0.05, ^##^*P* < 0.01 versus EAE group.

IL-6, TGF- β, IL-17A, and retinoic acid receptor-related orphan receptor gamma-t (RORγt) are important cytokines to activate microglia and play a certain role in its function and phenotype maintenance. In our study, IL-6, TGF-β, IL-17A, and RORγt mRNA relative expression in CNS tissues of mice were significantly augmented in EAE mice compared with the mice of NC group. Emodin treatment attenuated mRNA relative expressions of IL-6, TGF-β, IL-17A, and RORγt in EAE mice significantly (*P* < 0.05, Figures 9e,f,g,h). This suggested that high-dose emodin can inhibit transcription of inflammatory factors and inhibit the activation of microglia at the peak of EAE, thereby reducing inflammatory injury.

## Emodin inhibits microglia activation by inhibiting MyD88 activation

**Figure 10.**
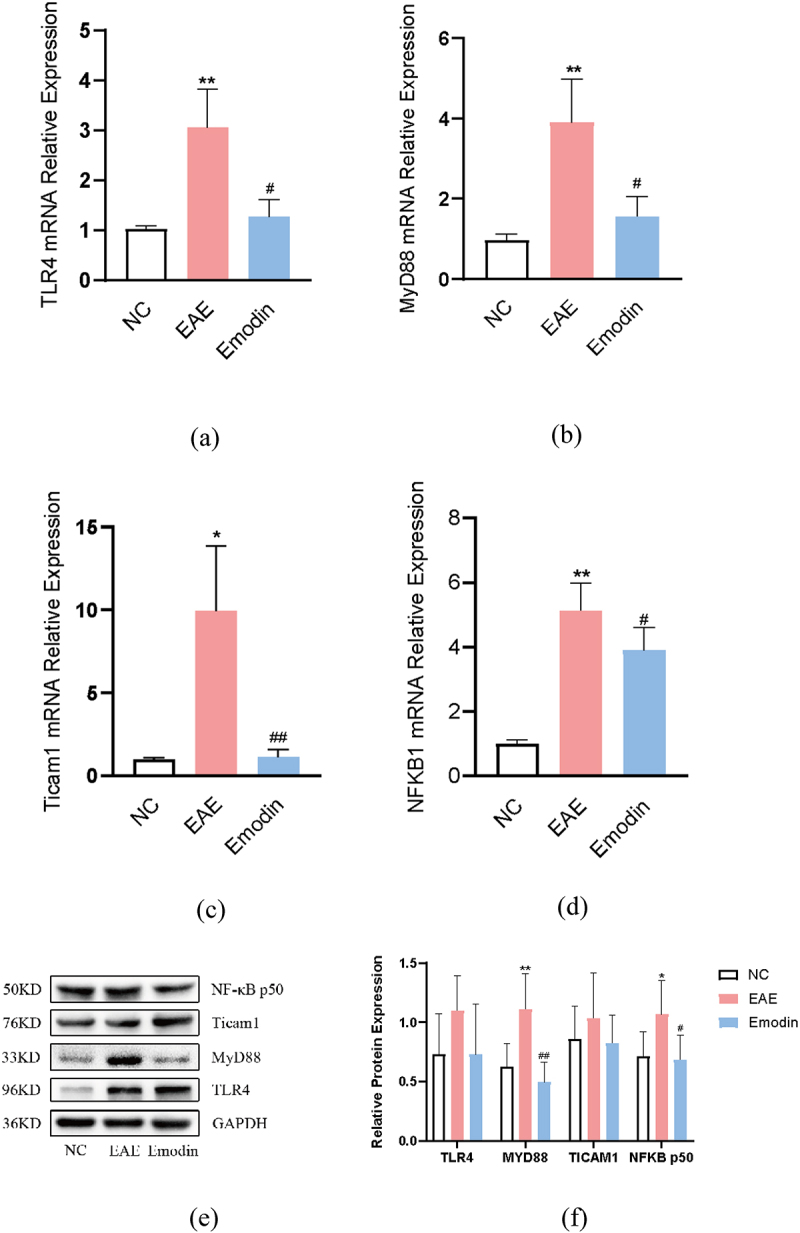
The effects of emodin on TLR4, Myd88, Ticam1, and NFKB1 mRNA relative expression and protein expression. Mice were sacrificed on 21 dpi. The cerebral cortex tissues are collected. The relative mRNA relative expression of TLR4 (a), Myd88 (b), Ticam1 (c), and NFKB1(d) was analyzed by qRT-PCR. The protein expression of TLR4, Myd88, Ticam1, and NFKB1 was analyzed by western blot. All data were expressed as mean ± S.D (n = 6). *P < 0.05, **P < 0.01 versus NC group, ^#^*P* < 0.05, ^##^*P* < 0.01 versus EAE group. Comparisons among each group were analyzed by one-way ANOVA in A, B, C, D, and F.

In order to analyze the molecular mechanism of high-dose emodin on the activation of microglia in EAE mice, we detected the relative expression levels of TLR4, MyD88, ticam1, and NFKB1 mRNA and protein by PCR and western blot, respectively. All of them were notably increased in EAE mice compared with the mice of NC group (*P* < 0.05, Figures 10). Emodin significantly reduced the TLR4 (Figures 10a), Myd88 (Figures 10b), Ticam1 (Figures 10c) and NFKB1 (Figures 10d) mRNA expressions in EAE mice (*P* < 0.05). For protein expression, higher Myd88 and NFKB1 were seen in EAE mice than those of NC group (*P* < 0.05, Figures 10e,f) and emodin diminished them significantly. However, there were no significant changes in the protein expression of TLR4 and Ticam1 among the three groups. Myd88 is one of TIR domain-containing adaptor molecules can be triggered by TLRs. The results indicate that emodin can targeting inhibit the activity of key adapter molecule Myd88, thereby inhibiting the signal transduction of TLR signalling pathway, alleviating the downstream NF- κB mediated inflammatory reaction, and alleviating the symptoms of EAE.

## Discussion

Emodin is an anthraquinone derivative having comprehensive anti-inflammatory, anti-cancer, and immunomodulatory effects, and it is widely used in the treatment of inflammatory, tumor, and immune system diseases [15,16]. However, none of the clinical or experimental studies have explored whether emodin exerts a therapeutic effect on EAE/MS. The present study is the first to confirm the efficacy of emodin in EAE through animal experiments by using mouse models. The results showed that emodin ameliorated the clinical symptoms in the acute phase of EAE mice at 21dpi. HE staining showed that emodin treatment decreased inflammatory infiltration in the brain and spinal cord of EAE mice. Immunohistochemistry results showed that the levels of MBP and BDNF increased significantly (*P* < 0.05) following the treatment. This finding suggests that emodin exerted certain anti-inflammatory and myelin protective effects on EAE mice.

To further explore the mechanism of action of emodin in EAE/MS treatment, we used network pharmacology to predict the targets and pathways. We found 42 common target genes including the core targets such as AKT1, ESR1, CASP3, STAT3, EGFR, CTNNB1, SRC, CDH1, FOS, and ANXA5. KEGG enrichment analysis showed that the top five KEGG pathways with high counts included pathways in cancer, prolactin signalling pathway, EGFR tyrosine kinase inhibitor resistance, hepatitis B pathway, PI3K–Akt signalling pathway (Figures 6b). Further analysis of these pathways showed that AKT1 is a key gene associated with the aforementioned pathways. Thus, emodin may ameliorate EAE/MS by reducing inflammation and by participating in the cell differentiation process, and PI3K/Akt may be the main pathway. AKT1 is one of the three members of the AKT serine-threonine protein kinase family. Akt is activated after its phosphorylation by PI3K, and activated Akt can further phosphorylate various downstream target genes associated with its biological role. ATK can activate IKB kinase (IKKα), thus causing the degradation of the NF-κB inhibitor IκB and resulting in NF-κB release from the cytoplasm, its nuclear translocation, and activation of its target genes. Studies have shown that the activated NF-κB1, also known as P50, is a key transcription regulator in the lipopolysaccharide (LPS)-induced microglia model, which activates pro-inflammatory cytokines [43,44]. In our study, we first performed molecular docking experiments on the key proteins in the PI3K/Akt pathway and found that emodin was well bound to PI3K, AKT1, and NF-κB1. Western blotting showed that emodin could significantly inhibit the phosphorylation levels of PI3K and AKT1. In addition, studies have shown that excessive activation of microglia occurred in both EAE mice and LPS-stimulated BV-2 cells. AKT and PI3K phosphorylation were found in the EAE model. The PI3K/Akt signalling pathway has been reported to be involved in inflammation regulation in the microglia [45–47]. This suggests that emodin may ameliorate EAE/MS by reducing inflammation and participating in the cell differentiation process via PI3K/AKT/NF-κB signalling pathway inhibition.

AKT1 regulation plays a crucial role in EAE. Ouyang et al. [48] reported that AKT1 knockout could enhance susceptibility to EAE in a T cell-intrinsic manner. Zenke et al. [49] reported that AKT1 could play a suppressive role in TLR4-mediated cytokine production. It suppressed both MyD88-dependent and TRIF-dependent TLR4 signalling independent of its kinase activity. Additionally, AKT1 regulates the microglial biological function including pro- and anti-inflammatory cytokine production, phagocytosis, autophagy, and homeostasis determined by apoptosis and metabolism. Our study results showed that the p-PI3K, p-Akt, and NF-κB levels increased significantly at 21 dpi in the EAE model, suggesting that PI3K/Akt signalling pathway activation is involved in the pathological process. Moreover, the secretion of activated microglia markers such as CD86 and CD206 increased significantly. Interestingly, after emodin administration, the activation of microglia and PI3K/Akt signalling pathways decreased simultaneously in the EAE mice. Thus, the potential action mechanism of emodin in EAE/MS treatment is microglial function regulation.

Studies have reported that the role of innate immunity in EAE/MS pathogenesis is as important as the adaptive immune response mediated by CD4^+^ T cells, and innate immunity plays a double-sided role in different stages throughout the disease process [50,51]. Myelin-reactive CD4^+^ T cells are activated in the periphery, and they infiltrate the CNS by secreting cytokines and chemokines and initiate an inflammatory cascade in EAE/MS [52]. Microglia are the resident macrophages of CNS that have important physiological functions in maintaining tissue homeostasis. Microglia can switch into diverse reactive phenotypes with neuroinflammation and neuroprotective function with changes in the microenvironment [53]. Microglial activation occurs before the occurrence of motor deficits [54]. In the acute phase of EAE/MS, microglia are activated into the phenotypes having binary states, which is probably a dynamic spectrum. Microglia are polarized to the M1 or M2 phenotype at any time in response to different stimuli. As the disease progresses, an overabundance of inflammatory cytokines skews microglial polarization toward the M1 phenotype [55,56]. These changes often cause negative effects such as cell phagocytosis, oxidative damage, antigen presentation, and T-cell costimulatory signal activation on myelin tissues. Hence, microglia are a target of EAE/MS treatment. Li et al. used exosomes derived from bone marrow mesenchymal stem cells for EAE treatment. The results showed that exosomes inhibited IL-6 released by M1 microglia and reduced the severity of inflammation and demyelination [57]. Schampel et al. [58] reported that nimodipine can reduce the inflammatory response and promote remyelination by inhibiting the activity of M1 microglia. Pro-inflammatory cytokines including Th17 that are produced by glial cells in the CNS or reach the CNS from the circulation also regulate the activity of microglia [59]. Th17 cells contribute to the pathology of MS. They traffic into CNS and secrete IL-17A. RORγt is featured by Th17 cells, and Th17 participates in controlling the development of EAE/MS [4,52,60–63]. It suggests that emodin may ameliorate EAE by reducing the infiltration of peripheral inflammatory cells into the CNS.

Many signalling pathways are involved in the activation of microglia, among which TLR4 signalling is the most important pathway. TLR4 mediates T-cell immunity by stimulating microglia to express MHC-II and related costimulatory molecules. On the other hand, it can activate downstream inflammation and apoptosis pathways, thus activating the inflammatory cascade reactions of the CNS [64]. TLR4 mainly depends on MyD88 and Ticam1 proteins to activate downstream signals. It can stimulate the nuclear translocation of NF-κB, promote the release of inflammatory factors such as IL-1β, IL-6, IL-17, and TNF-α, and cause tissue damage or activate autophagy or apoptosis induced by PIK3/AKT/mTOR pathways [65,66]. The TLR4 signalling pathway is involved in the pathogenesis of EAE/MS and mediates the activation of M1 microglia. LPS combined with IFN-γ, inducing TLR4, can highly induce the polarization of microglia to the pro-inflammatory type via its adaptor protein tram1 and promote the expression of IL-6, IL-1β, and iNOS. In this process, IκB, p65, p50, and nuclear translocation of NF-κB play important roles [67,68]. Many studies have reported that the inhibition of the TLR4 receptor or its ligand may improve or delay the pathological process [69]. For example, MyD88 knockout mice were almost completely immune to EAE induced by MOG; the severity of symptoms and inflammatory response in the mice treated with MyD88 blocker were significantly lower than those in the control group [70,71]. The release of interleukin and cytokines was inhibited with the downregulation of TRIF expression, thereby reducing the severity of EAE [72]. To further clarify the mechanism underlying the regulation of microglia differentiation by emodin, we verified its classical upstream activation pathway. We found that the mRNA transcription levels of TLR4, MyD88, Ticam1, and NFKB1 were significantly increased in the brain of EAE mice. Emodin treatment could significantly downregulate the transcription level of this signalling pathway. Quantification of proteins indicated that MyD88 was significantly upregulated in the EAE model, and the activity of MyD88 was significantly inhibited after emodin treatment. Taken together, emodin may inhibit the activity of MyD88 and block transmission of the TLR signalling pathway to limit the proliferation of immune cells, suppress inflammatory responses induced by PI3K/Akt/NF-κB in microglia, and improve the clinical symptoms of EAE/MS.

## Conclusion

**Figure 11.**
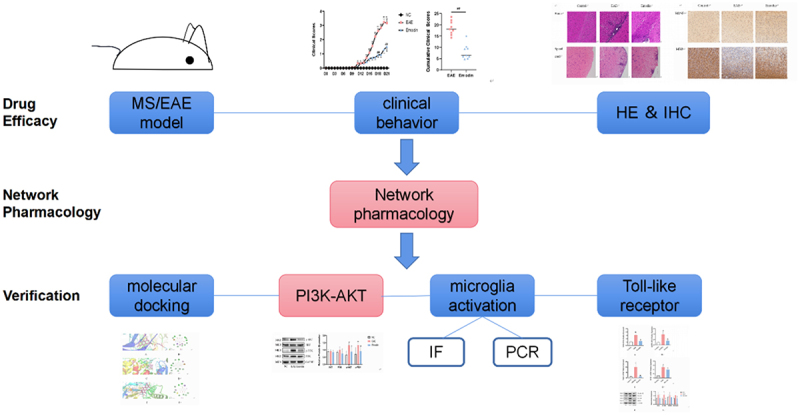
Schematic diagram of potential mechanisms linking the emodin with MS.

The finding indicated protective effects of emodin on EAE by inhibiting the microglia activation and inflammation via Myd88/PI3K/Akt/NF-κB signalling pathway (Figures 11). Emodin may improve EAE by affecting the infiltration of peripheral inflammatory cells into the CNS. We used the network pharmacology analysis to explore the potential targets and the action mechanism of emodin in MS/EAE treatment. The KEGG analysis showed that PI3K/AKT1/NF-κB may be an important pathway mediating the effect of emodin on EAE/MS. Molecular docking and animal experiments confirmed that emodin has good binding properties with the aforementioned proteins and that it significantly inhibits the activation of the PI3K/AKT1/NF-κB signalling pathway. Further exploration of the molecular mechanism indicated that the therapeutic effect of emodin on EAE is mediated via MyD88 activation and inhibition of the down transmission of TLR signals to reduce the activation of microglia and its induced inflammatory response. This study explored the complementary and alternative therapies for EAE/MS in the acute stage. The results provide an experimental reference for MS treatment with natural products. However, the role of emodin in the chronic stage and action mechanism remains to be further investigated.
